# Dual Effects of Cell Free Supernatants from *Lactobacillus acidophilus* and *Lactobacillus rhamnosus* GG in Regulation of MMP-9 by Up-Regulating *TIMP-1* and Down-Regulating CD147 in PMA- Differentiated THP-1 Cells

**DOI:** 10.22074/cellj.2018.4447

**Published:** 2017-11-04

**Authors:** Faezeh Maghsood, Abbas Mirshafiey, Mohadese M. Farahani, Mohammad Hossein Modarressi, Parvaneh Jafari, Elahe Motevaseli

**Affiliations:** 1 Department of Immunology, School of Public Health, Tehran University of Medical Sciences, Tehran, Iran; 2 Department of Medical Genetics, School of Medicine, Tehran University of Medical Sciences, Tehran, Iran; 3 Department of Microbiology, Science Faculty, Islamic Azad University, Arak Branch, Arak, Iran; 4 Department of Molecular Medicine, School of Advanced Technologies in Medicine, Tehran University of Medical Sciences, Tehran, Iran; 5 Food Microbiology Research Center, Tehran University of Medical Sciences, Tehran, Iran

**Keywords:** CD147, Inflammation, MMP, Probiotics, TIMP

## Abstract

**Objective:**

Recent studies have reported dysregulated expression of matrix metalloproteinases (MMPs), especially MMP-2, MMP-9, tissue inhibitor of metalloproteinase-1, -2 (*TIMP-1, TIMP-2*), and extracellular matrix metalloproteinase inducer (EMMPRIN/CD147) in activated macrophages of patients with inflammatory diseases. Therefore, MMP-2, MMP-9, and their regulators may represent a new target for treatment of inflammatory diseases. Probiotics, which are comprised of lactic acid bacteria, have the potential to modulate inflammatory responses. In this experimental study, we investigated the anti-inflammatory effects of cell-free supernatants (CFS) from Lactobacillus acidophilus (*L. acidophilus*) and *L. rhamnosus* GG (LGG) in phorbol myristate acetate (PMA)-differentiated THP-1 cells.

**Materials and Methods:**

In this experimental study, PMA-differentiated THP-1 cells were treated with CFS from L. acidophilus, LGG and uninoculated bacterial growth media (as a control). The expression of *MMP-2, MMP-9, TIMP-1*, and *TIMP-2* mRNAs were determined using real-time quantitative reverse transcription polymerase chain reaction (RT- PCR). The levels of cellular surface expression of CD147 were assessed by flow cytometry, and the gelatinolytic activity of MMP-2 and MMP-9 were determined by zymography.

**Results:**

Our results showed that CFS from both *L. acidophilus* and LGG significantly inhibited the gene expression of *MMP-9* (P=0.0011 and P=0.0005, respectively), increased the expression of *TIMP-*1 (P<0.0001), decreased the cell surface expression of CD147 (P=0.0307 and P=0.0054, respectively), and inhibited the gelatinolytic activity of MMP-9 (P=0.0003 and P<0.0001, respectively) in PMA-differentiated THP-1 cells. Although, MMP-2 expression and activity and TIMP-2 expression remained unchanged.

**Conclusion:**

Our results indicate that CFS from *L. acidophilus* and LGG possess anti-inflammatory properties and can modulate the inflammatory response.

## Introduction

Inflammation is a protective response to infections and tissue damage. However, dysregulation of inflammatory responses can prolong immune responses and inflammation, leading to inflammatory diseases (1, 2). A class of dysregulated enzymes during inflammation is matrix metalloproteinases (MMPs). MMPs are a family of endopeptidases that bind to macromolecules from extracellular matrix and play an important role in physiological and pathological tissue remodeling (3). MMP-2 and MMP-9 are the major MMPs secreted by inflammatory activated macrophages (4, 5). Therefore, they are increased in almost all inflammatory diseases, such as osteoarthritis (6), inflammatory cardiomyopathy (7-9), rheumatoid arthritis (3), systemic lupus erythematosus and diabetes mellitus (10-12). Expression and activity of MMPs are regulated by the tissue inhibitors of metalloproteinases (TIMPs) and extracellular matrix metalloproteinase inducer (EMMPRIN/CD147) secreted by different types of cells, including inflammatory activated macrophages (13). However, the expression and activity of these genes are dysregulated in mentioned diseases (9, 14-18). Therefore, MMP-2 and MMP-9 and their regulators are attractive targets for preventing and improving inflammatory diseases.

THP-1 is a human monocytic cell line. After treatment with phorbol esters, differentiated THP-I cells act more like natural monocyte-derived macrophages in comparison with other human myeloid cell lines, such as human promyelocytic *leukemia cells* (HL-60), U937, KG-1, or human erythroleukaemia (HEL) cell lines (19). Phorbol 12-myristate 13-acetate (PMA) is a member of the phorbol esters family. Phorbol esters, which are analogues of diacylglycerol (DAG), interact with the DAG-binding site and activate most protein kinase C isozymes (20). On the other hand, protein kinase C pathways are involved in the maturation of THP-1 cells with PMA (21). Thus, PMA differentiated THP-1 cell line is a representative macrophage model, *in vitro*.

Gut microbiota is critical for modulation of innate and adaptive immune systems. Imbalance of gut microbiota results in the loss of immune-regulation, overgrowth of pathogenic microorganisms, and increased inflammation (22). Gut microbiota in patients with inflammatory diseases, such as inflammatory bowel disease, allergic inflammation, diabetes mellitus, multiple sclerosis, psoriasis, rheumatoid arthritis, and atherosclerosis, often differs from that of healthy people (1, 22-25). Different species of probiotic lactobacilli and bifidobacteria reduce inflammatory mediators and lesions, in vitro and in experimental models and patients (26-29). Recent studies have indicated that the secreted components of probiotic bacteria can reduce inflammatory responses (30, 31). Although there is some evidence confirming the potential role of probiotic supernatants in decreasing inflammatory cytokines and mediators, further research is required to evaluate their effect on the modulation of MMP-2 and MMP-9 and their regulators.

In the present study, we investigated the ability of cellfree supernatants (CFS) from two *Lactobacillus (L.)* sp., *L. acidophilus* and *L. rhamnosus* GG (LGG), to decrease *MMP-2* and *MMP-9* expression and activity using a PMA-induced cell differentiation model of the human monocytic cell line, THP-1, *in vitro*. We also examined the ability of CFS from *L. acidophilus* and LGG to modulate the expression of *TIMP-1, TIMP-2* and CD147, which are involved in the regulation of *MMP-2* and *MMP-9* expression and activity.

## Materials and Methods

This experimental study was approved by the Ethical Committee of Tehran University of Medical Sciences, Tehran, Iran (IR.TUMS.REC.1395.2816).

### Sources of cell line and reagents

Human monocytic THP-1 cells were obtained from the Pasteur Institute, National Cell Bank of Iran (NCBI), Tehran, Iran. All reagents used for cell culture, including Roswell Park Memorial Institute (RPMI) 1640 medium, fetal bovine serum (FBS), L-glutamine, penicillin and streptomycin were purchased from Gibco (Germany). PMA, dimethylsulfoxide (DMSO), Coomassie blue and 3-(4,5-Dimethylthiazol-2-yl)-2,5-diphenyltetrazolium bromide (MTT) were purchased from Sigma-Aldrich Company (Germany). RNA Extraction kit was obtained from YTA (Iran), while PrimeScript RT-PCR Kit and SYBR Premix Ex Taq were purchased from Takara Bio (Japan). Phycoerythrin-labeled mouse anti-human CD147 antibodies and IgG1 antibodies were obtained from the eBioscience Corporation. The primers of target genes and glyceraldehyde 3-phosphate dehydrogenase (*GAPDH*) used for real-time quantitative reverse transcriptionpolymerase chain reaction (RT-PCR) were synthesized and purified by Bioneer (Germany). List of primer sets are available in the Table 1.

**Table 1:** Sequence of the primers applied for real-time quantitative RT-PCR


Gene	Primer sequencing (5ˊ-3ˊ)

MMP-2	F: GGCAGTGCAATACCTGAACACC
	R: GTCTGGGGCAGTCCAAAGAACT
MMP-9	F: GCACGACGTCTTCCAGTACC
	R: CAGGATGTCATAGGTCACGTAGC
TIMP-1	F: TTCTGGCATCCTGTTGTTGCT
	R: CCTGATGACGAGGTCGGAATT
TIMP-2	F: TGGAAACGACATTTATGGCAACCC
	R: CTCCAACGTCCAGCGAGACC
GAPDH	F: GAGTCCACTGGCGTCTTCA
	R: TCCTTGAGGCTGTTGTCATACTTC


RT-PCR; Reverse transcription polymerase chain reaction, MMPs; Matrix metalloproteinases, GAPDH; Glyceraldehyde 3-phosphate dehydrogenase, and TIMPs; Tissue inhibitors of metalloproteinases.

### THP-1 cell culture and differentiation

Human monocytic THP-1 cells were cultured in RPMI- 1640 medium supplemented with 10% heat inactivated FBS, 2 mM L-glutamine, 100 U/ml penicillin, 100 μg/ml streptomycin, and incubated at 37˚C, 5% CO_2_ and 95% humidity. The medium was changed daily, and cells were passaged weekly. After proliferation, 1×10^6^ cells were centrifuged (1000×g for 5 minutes) and then seeded. The treated cells with PMA (final concentration of 50 ng/ml) in RPMI-1640 medium supplemented with 10% FBS for 24 hours were differentiated into activated THP-1 cells.

Subsequently, the culture medium was aspirated to remove nonadherent cells, and attached cells were washed with RPMI-1640 medium, three times. PMAdifferentiated THP-1 cells were separated, centrifuged (1000×g for 5 minutes), and seeded at a density of 1×10^6^ cells/ml into six-well culture plates in RPMI-1640 with 10% FBS to allow for cell adherence for 24 hours.

### Preparation of supernatants from lactobacillus cultures and treatment of THP-1 cells

*L. rhamnosus* strain LGG (LbR) and *L. acidophilus* strain La5 (LbA) were inoculated separately in de Man Rogosa Sharpe (MRS) broth medium (pH=6.5, Merck, Germany), containing a rich nutrient base, polysorbate, acetate, magnesium and manganese, to enhance the growth and proliferation of lactobacilli. Following incubation at 37˚C for 48 hours under microaerophilic conditions, bacterial cultures reached an optical density (OD) of 0.7 to 0.8 at 600 nm, which complies with bacterial numbers of approximately 10^9^ cfu/ml, as determined by plate counting on MRS agar for *L.* sp. Bacterial cultures were centrifuged at 1100×g for 15 minutes at 4˚C and filtered through a 0.2 μm membrane filter to remove the remaining bacteria and debris. The pH of CFS was decreased from 6.5 (MRS broth pH) to 4.4 ± 0.2. Noninoculatad MRS broth adjusted to pH between LAS (LbA supernatant) pH and LRS (LbR supernatant) pH with lactate (called MRL) was used to test whether lactate produced by L. acidophilus and LGG, while pH change would affect tests. Four different treatments were performed for 24 hours, as follows: LAS (pH=4.5, 15% v/v), LRS (pH=4.23, 10% v/v), MRS (pH=6.5, 15% v/v), and MRS adjusted with lactate (MRL, pH=4.35, 10% v/v).

### MTT assay

Cytotoxicity was measured using MTT assay. PMA activated cells were seeded at a density of 5×10^4^ cells into 96-well culture plates in RPMI-1640 medium with 10% FBS, and incubated to recover and adhere for 24 hours. Subsequently, cells were treated for 24 hours with 1, 2, 5, 10, 15, 20 and 50% (v/v) lactobacilli culture supernatants, MRL and MRS. Plates were incubated in 5% CO_2_ at 37˚C in a humidified incubator. The medium was replaced and 20 μl of MTT solution [5 mg/ml in phosphate-buffered saline (PBS)] was added to each well, while plates were incubated for 4 hours at 37˚C in a humidified incubator. The supernatant was carefully aspirated, and 100 μl of DMSO was added to each well to solubilize formazan blue crystals. Following an incubation of 15 minutes, absorbance at 570 nm was measured using an Absorbance Reader (Biotek, Absorbance Microplate Reader, USA) according to the manufacturers’ instructions. Cell viability was determined as follows: viability (percentage of control)=[(absorbance sample-absorbance blank)/(absorbance control-absorbance blank)]×100.

### RNA extraction, cDNA synthesis and real-time quantitative reverse transcription polymerase chain reaction

THP-1 cells were differentiated and treated as previously described. A total RNA extraction kit (YTA, Iran) was used to extract total RNA from treated cells according to the manufacturers’ instructions. RNA concentration and purity were assessed spectrophotometrically from the ratio of absorbance at 260 nm and 280 nm using a Nanodrop 2000c spectrophotometer (Thermo Fisher Scientific, Canada) in molecular-grade water. Synthesis of cDNA from the isolated total RNA was conducted using the PrimeScript RT reagent Kit (Takara Bio, Japan).

In brief, 5xPrimeScriptTMBuffer (2 μl), PrimeScript RT Enzyme Mix 1(0.5 μl), oligo dt Primer (0.5 μl) and Random 6 mers (0.5 μl) were added to 1 μg RNA from each sample, the reaction volume was brought to 10 μl with RNase free water, mixed gently, and incubated at 37˚C for 15 minutes to activate the reverse transcriptase enzyme and 85˚C for 5 seconds to inactivate the reaction.

After reverse transcription, cDNA was used for realtime quantitative RT-PCR on ABI-7000 Detection System thermal cycler (Applied Biosystems, USA) using SYBR Premix Ex Taq (Takara Bio, Japan). The RT-PCR was performed in a final volume of 20 μl containing 10 μl SYBR green master mix, 4 μl cDNA, 2 μl each forward and reverse primer (10 μM), and 2 μl nuclease-free water. Thermal cycling conditions for all genes were as follows: template pre-denaturation (30 seconds at 95˚C), denaturation (5 seconds at 95˚C), annealing and extension (30 seconds at 60˚C) for 50 cycles. The protocol for melting curve analysis was as follows: 15 seconds at 95˚C, 1 minute at 60˚C, and 15 seconds at 95˚C. Experiments were performed in duplicate for each data point. *GAPDH* mRNA was amplified as a housekeeping gene, and fold changes in each target mRNA expression relative to *GAPDH* were calculated by the 2^−ΔΔCT^ method. Expression of mRNA is defined as the change in mRNA copy numbers relative to positive control cells (PMAdifferentiated THP-1 cells).

### Gelatin zymography

To determine the effect of bacterial CFS on gelatinolytic activity of MMP-2 and MMP-9 by gelatin zymography, THP-1 cells were differentiated and treated as previously described. The conditioned medium was collected and centrifuged (1000 g×10 minutes) to remove debris. This technique was performed using 10% polyacrylamide/ sodium dodecyl sulfate (SDS) gels with 0.1% (w/v) gelatin. In brief, equal amounts of protein (10 μg) from each treatment (adjusted by Bradford assay) were diluted with 5 μl of 6× sample buffer (without prior boiling), incubated at room temperature for 15 minutes, and 20 μl of samples was loaded to each lane. After electrophoresis, gels were washed three times in 50 ml of 2.5% Triton X-100 at room temperature for 30 minutes to remove SDS and allow proteins to renature, immersed in development buffer (50 mM Tris-HCl, 5 mM CaCl_2_, 0.01% NaN3, 1 μM ZnCl_2_, and 200 mM NaCl, pH=7.5) at room temperature for 15 minutes, and then incubated overnight at 37˚C in the same buffer to activate enzymes to digest the gelatin substrate. Subsequently, gels were rinsed briefly with water, stained with 0.5% Coomassie blue in 30% methanol and 10% acetic acid for 2 hours, and destained in a solution of 30% methanol and 10% acetic acid until clear bands indicating gelatinolytic activity appeared against a blue background of undigested gelatin. Using the Bio Rad GS-800 Calibrated Densitometer (Bio Rad, USA), gels were scanned and intensity of bands was determined by Image J software (1.46r).

### Flow cytometry

To quantify the effect of CFSs on cell surface expression of CD147, THP-1 cells were differentiated and treated as previously described. Cells were then separated by ice cold PBS, centrifuged, resuspended and divided into two tubes. According to the manufacturers’ instructions, one group of tubes was treated with phycoerythrin-labeled mouse anti-human CD147 antibodies (5 μl), and another group of tubes was treated with phycoerythrin-labeled mouse IgG1 antibodies (5 μl) for 45 minutes at 4˚C, as a control. The cell surface expression of CD147 was quantified using FACS Calibur ﬂowcyto meter (Becton Dickinson, Germany). FlowJo software (7.6.1) was used to analyze the FACS data and calculate mean fluorescence intensity (MFI).

### Statistical analysis

GraphPad Prism 6.0 software was used for all statistical analysis. All data were expressed as a mean ± SEM of three separate experiments. P<0.05 was considered statistically significant. Statistical differences among groups were determined using one-way analysis of variance (ANOVA). Dunnetts’ adjustment was used for multiple comparisons.

## Results

### Differentiation of THP-1 cells

**Fig.1:**
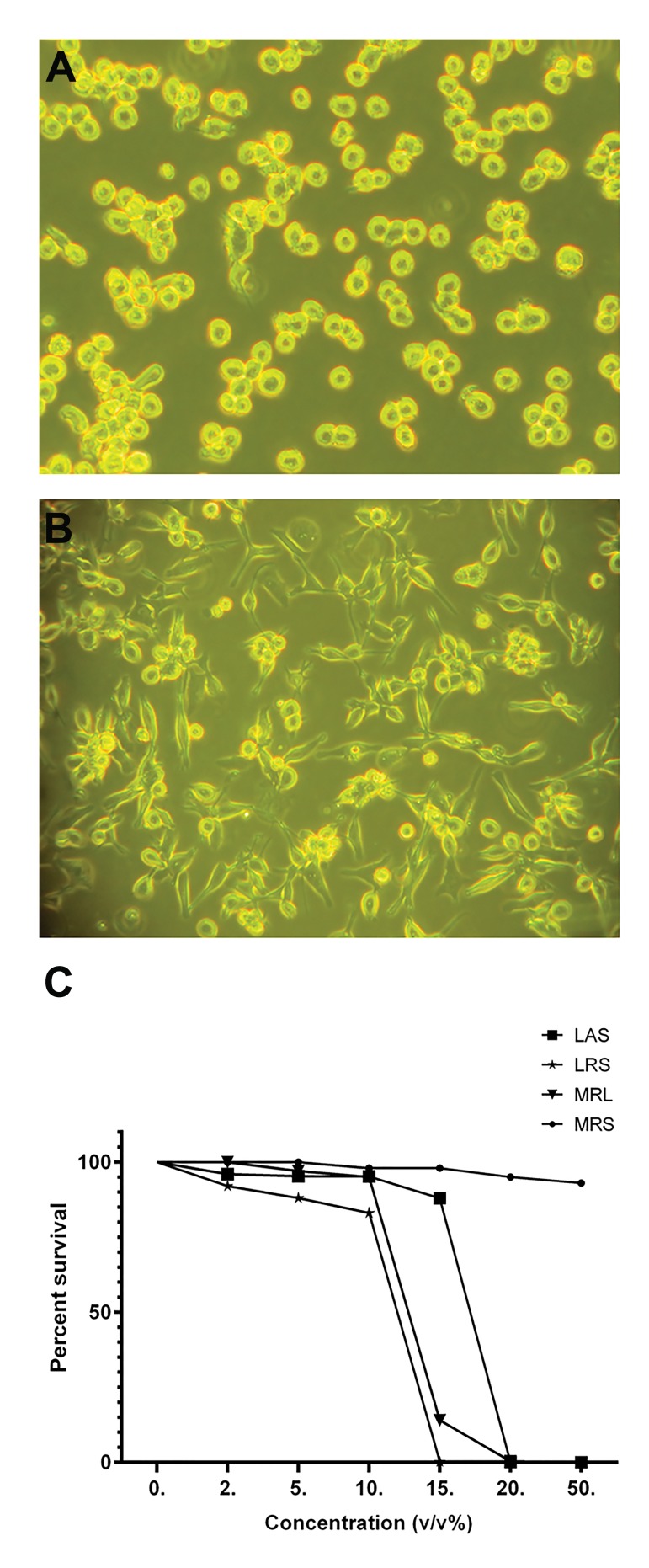
Undifferentiated and PMA-differentiated THP-1 cells and the effect of lactobacilli supernatant on cell viability. A. Undifferentiated THP- 1 cells with round phenotype, B. Induction of monocyte-macrophage differentiation by PMA (50 ng/ml) after 24 hours accompanied by the adherence of cells to the surface of plates with amoeboid-like phenotype, and C. Effects of different concentrations of LAS, LRS, MRL and MRS (0, 2, 5, 10, 15, 20 and 50 % v/v) on the viability of PMA-differentiated THP-1 cells were determined using MTT assay. LAS; *Lactobacillus acidophilus* supernatant, LRS; *Lactobacillus rhamnosus* GG supernatant, MRS; De Man Rogosa Sharpe, MRL; MRS with lactic acid, MTT; 3-(4,5-Dimethylthiazol-2-yl)-2,5-diphenyltetrazolium bromide, and PMA; Phorbol myristate acetate.

THP-1 cells were cultured at a density of 1×10^6^ cells/ml in six-well culture plates in RPMI-1640 with 10% FBS. All cells were treated with PMA (final concentration of 50 ng/ml), except those in the negative control group. After 24 hours, cells were evaluated using a microscope (Olympus, USA). More than 80% of PMA-activated THP-1 cells were flatted and adhered to the plastic as macrophage-like cells, indicating differentiation and successful establishment of the model (Fig .1A, B).

### Inhibition of Phorbol 12-myristate 13-acetate-differentiated THP-1 cells proliferation by Lactobacillus *acidophilus supernatant* and *Lactobacillus rhamnosus* GG supernatant dependent on lactate and acidity

Cell growth inhibition was measured by MTT assay. The IC_50_ values (concentration giving half-maximal inhibition) of LAS and LRS against PMA-differentiated THP-1 cells were 17 and 14% (v/v), respectively, while the value of 15 and 10% (v/v) were used for treatment with LAS and LRS, respectively. The selected concentrations exerted 80-90% cell viability on PMA-differentiated THP-1 cells. The effects of LAS and LRS on PMA-differentiated THP-1 cells were equal to those of MRL (MRS with pH adjusted to that of LAS and LRS) at similar concentrations (Fig .1C). It indicates that the inhibitory effect of LAS and LRS on PMA-differentiated THP-1 cells is lactate- and acidity-dependent. These results revealed that the main cause of PMA-differentiated THP-1 cell death was related to the acidity and lactate condition, not to other substances in the supernatant of the L.sp.

### Effect of *Lactobacillus acidophilus* supernatant and *Lactobacillus rhamnosus* GG supernatant on expression of *MMP-2* and *MMP-9* mRNA

**Fig.2:**
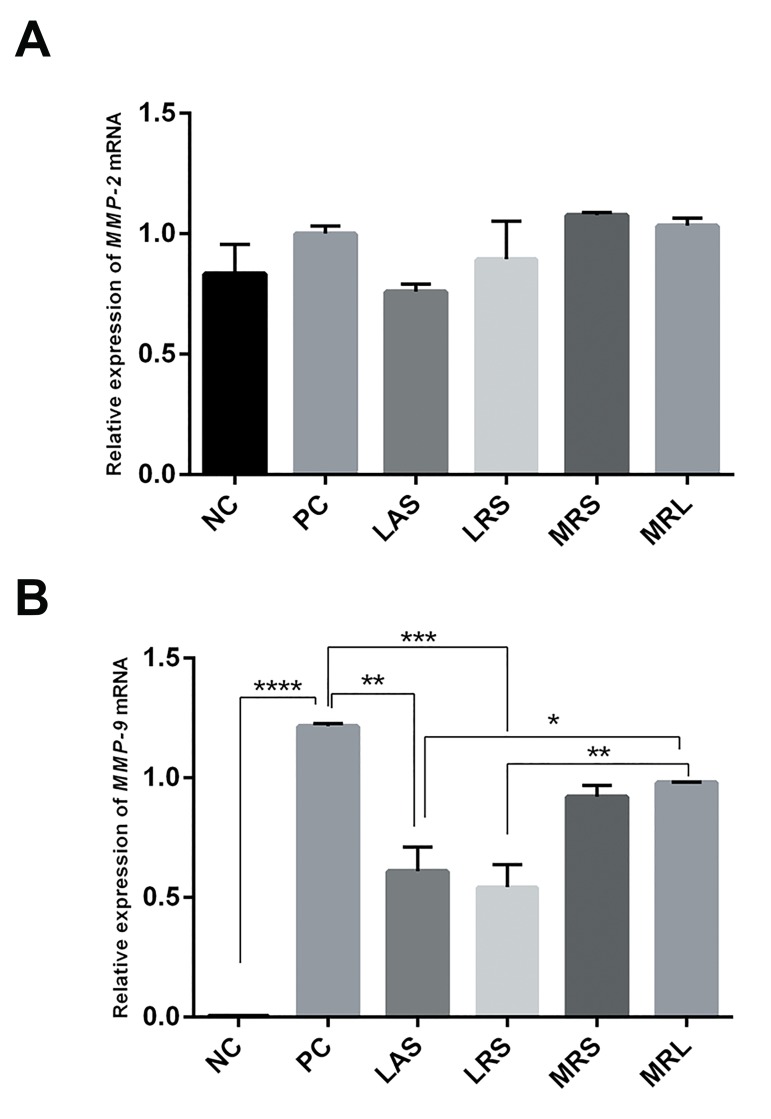
Effects of lactobacilli supernatant on expression of *MMP-2* and *MMP-9* mRNAs. A. Effects of LAS, LRS, MRL and MRS on expression of *MMP-2* mRNAs and B. Effects of LAS, LRS, MRL and MRS on expression of *MMP-9* mRNAs in PMA-differentiated THP-1 cells. Relative quantification of gene expression was performed by the 2^−ΔΔCt^ method. The results showed the means ± SEM from six independent experiments. Significant difference was compared with positive control: *; P<0.05, **; P<0.01, ***; P<0.001, ****; P<0.0001. NC; Negative control (undifferentiated THP-1 cells), PC; Positive control (PMA-differentiated THP-1 cells), LAS; *Lactobacillus acidophilus* supernatant, LRS; *Lactobacillus rhamnosus* GG supernatant, MRS; De Man Rogosa Sharpe, MRL; MRS with lactic acid, and MMPs; Matrix metalloproteinases.

*MMP-9* and *MMP-2* mRNA levels in PMA-differentiated THP-1 cells were measured by SYBR Green qPCR. Expression of *MMP-9* mRNAs was significantly higher in positive control than in negative control (NC: 0.003463 ± 0.00095, PC: 1.216 ± 0.01038, P<0.0001), whereas expression of MMP-2 mRNAs had no significant change (NC: 0.8325 ± 0.1236, PC: 1.001 ± 0.03127, P=0.6289). In addition, after 24 hours treatment with LRS, LAS, MRL, and MRS on PMA-differentiated THP-1 cells, LRS and LAS significantly down-regulated MMP-9 mRNA levels (0.5427 ± 0.09367, P=0.0005 and 0.6091 ± 0.1016, P=0.0011, respectively), whereas MRS and MRL alone had no significant effect (0.9207 ± 0.04713, P=0.1052 and 0.981 ± 0.0003, P=0.2289, respectively). By contrast, LRS and LAS did not reduce MMP-2 mRNA levels (0.8941 ± 0.157, P=0.8907 and 0.76 ± 0.03, P=0.4258, respectively) (Fig .2).

### Effect of *Lactobacillus acidophilus* supernatant and *Lactobacillus rhamnosus* GG supernatant on expression of *TIMP-1* and *TIMP-2* mRNA

**Fig.3:**
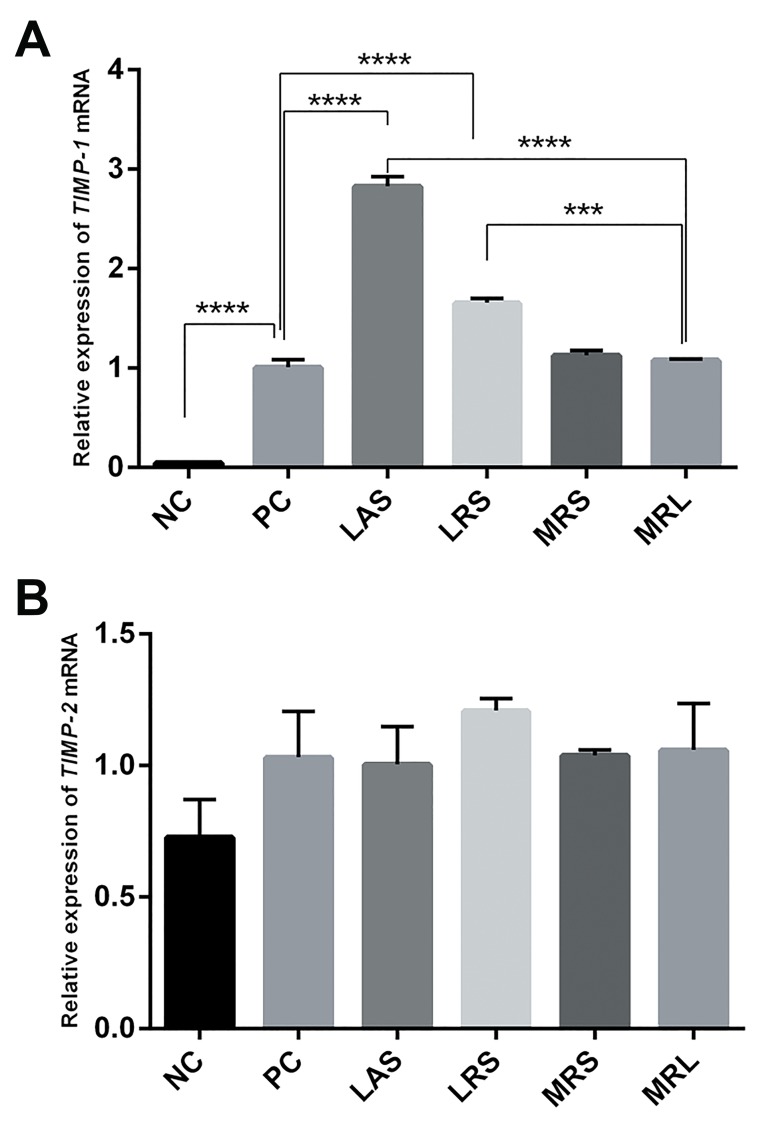
Effects of lactobacilli supernatant on expression of *TIMP-1* and *TIMP- 2* mRNAs. A. Effects of LAS, LRS, MRL and MRS on expression of TIMP-1 mRNAs and B. Effects of LAS, LRS, MRL and MRS on expression of TIMP-2 mRNAs in PMA-differentiated THP-1 cells. Relative quantification of gene expression was performed by the 2−ΔΔCt method. The results showed the means ± SEM from six independent experiments. Significant difference was compared with positive control: ***; P<0.001, ****; P<0.0001. NC; Negative control (undifferentiated THP-1 cells), PC; Positive control (PMA-differentiated THP-1 cells), LAS; *Lactobacillus acidophilus supernatant*, LRS; *Lactobacillus rhamnosus* GG supernatant, MRS; De Man Rogosa Sharpe, MRL; MRS with lactic acid, and MMPs; Matrix metalloproteinases.

Real-time quantitative RT-PCR showed that the expression of *TIMP-1* mRNAs was significantly higher in the positive control than in the negative control group (NC: 0.04525 ± 0.0093, PC: 1.007 ± 0.079, P<0.0001), while the expression of *TIMP-2* remained unchanged (NC: 0.7273 ± 0.1432, PC: 1.032 ± 0.1736, P=0.3867). In addition, after 24-hour incubation of PMA-differentiated THP-1 cells with LRS, LAS, MRL, and MRS, LRS and LAS up-regulated *TIMP- 1* mRNA levels (1.659 ± 0.04181, P<0.0001 and 2.829 ± 0.095, P<0.0001, respectively), whereas MRS and MRL alone had no significant effect (1.127 ± 0.05, P=0.4868 and 1.081 ± 0.009, P=0.8268, respectively). Furthermore, LRS and LAS could not affect *TIMP-2* mRNA levels (1.209 ± 0.04592, P=0.8083 and 1.004 ± 0.1434, P=0.9998, respectively) (Fig .3).

### Effect of *Lactobacillus acidophilus* supernatant and *Lactobacillus rhamnosus* GG supernatant on MMP-2 and MMP-9 activities

**Fig.4:**
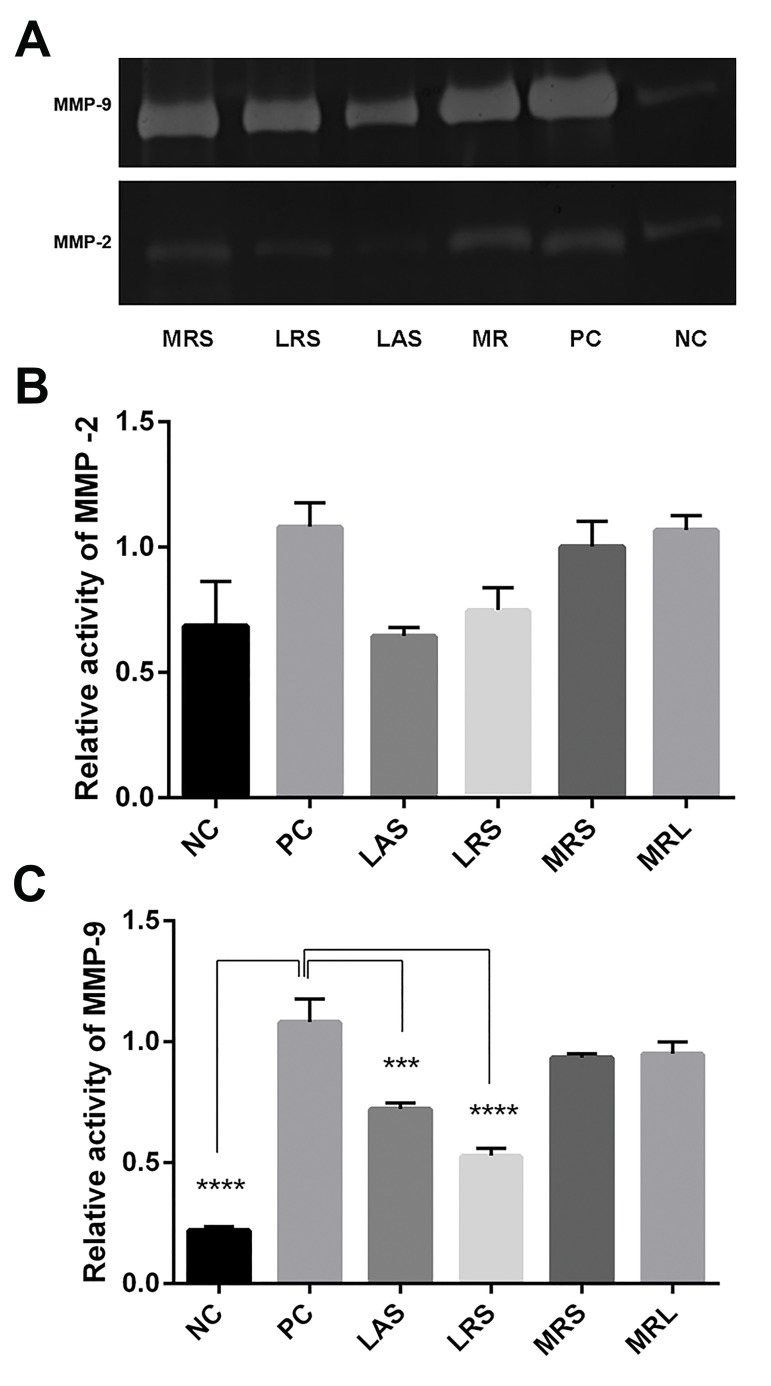
Effects of LAS, LRS, MRL and MRS on gelatinolytic activities of MMP- 2 and MMP-9. A. Representative gelatin zymography for MMP-2 and MMP-9, B. Relative activity of MMP-2, and C. Relative activity of MMP-9. The results showed the means ± SEM of three independent experiments. Significant difference was compared with positive control: ***; P<0.001, ****; P<0.0001. NC; Negative control (undifferentiated THP-1 cells), PC; Positive control (PMA-differentiated THP-1 cells), LAS; *Lactobacillus acidophilus* supernatant, LRS; *Lactobacillus rhamnosus* GG supernatant, MRS; De Man Rogosa Sharpe, and MRL; MRS with lactic acid, and MMPs; Matrix metalloproteinases.

Gelatin zymography was performed to compare MMP- 2 and MMP-9 activities between control and treated cells. MMP-9 activity was significantly higher in positive controls than in negative controls (NC: 0.2198 ± 0.01525, PC: 1.082 ± 0.094, P<0.0001). LAS and LRS significantly reduced MMP-9 activity (0.7222 ± 0.025, P=0.0003 and 0.5288 ± 0.03, P<0.0001, respectively) as compared to the positive controls. By contrast, LRS and LAS could not decrease MMP-2 activity (0.7493 ± 0.088, P=0.1904 and 0.6461 ± 0.03, P=0.0788, respectively, Fig .4).

### Effects of Lactobacillus acidophilus supernatant and Lactobacillus rhamnosus GG supernatant on CD147 expression

**Fig.5:**
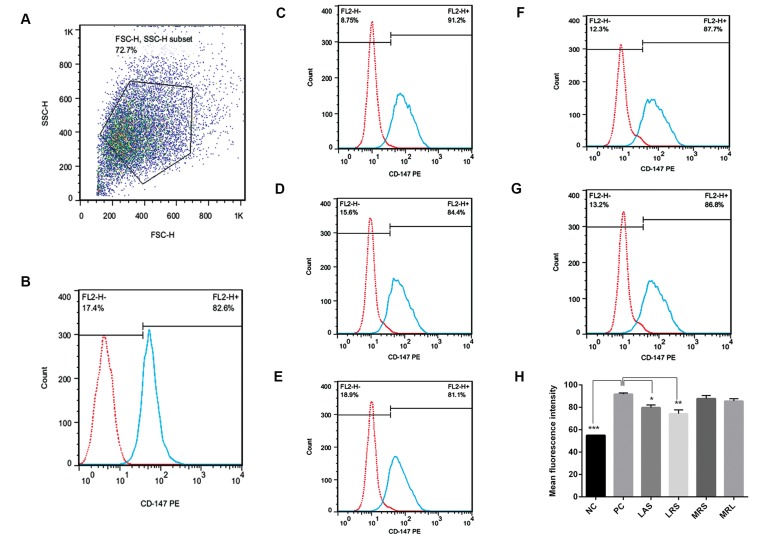
Effects of LAS, LRS, MRL and MRS on expression of CD147 protein in PMAdifferentiated THP-1 cells. Cell surface expression of CD147 was quantified after treatment with phycoerythrin-labeled mouse anti-human CD147 antibodies. A. Representative PMA-differentiated THP-1 flow cytometry scatter diagram for cells, B. NC (undifferentiated THP-1 cells), C. PC (PMA-differentiated THP-1 cells), D. LAS, E. LRS, F. MRS, G. MRL, and H. MFI of the six groups shown above. Data are represented as means± SE of 3 independent experiments. Significant difference was compared with positive control: *; P<0.05, **; P<0.01, ***; P<0.001. NC; Negative control, PMA; Phorbol myristate acetate, PC; Positive control, LAS; *Lactobacillus acidophilus* supernatant, LRS; *Lactobacillus rhamnosus* GG supernatant, MRS; De Man Rogosa Sharpe, MRL; MRS with lactic acid, and MFI; Mean fluorescence intensity.

Flow cytometry analysis indicated that CD147 was expressed at significantly higher levels in positive controls than in negative controls (NC: 54.95 ± 0.015, PC: 91.75 ± 1.15, P=0.0001). In addition, incubation of PMA-differentiated THP-1 cells with LAS and LRS significantly decreased CD147 expression (79.75 ± 2.35, P=0.0307 and 74.4 ± 3.3, P=0.0054, respectively) as compared to the positive control (Fig .5).

## Discussion

Tissue damage and destruction as the main pathogenesis of inflammatory diseases is mediated by MMPs through extracellular matrix degradation. Probiotics can help reduce inflammation. However, the effects of secreted components of probiotic bacteria on MMPs have not been established. Activated macrophages are one of the major sources of MMP-2 and MMP-9 during inflammation processes. In the present study, we chose PMAdifferentiated THP-1 cells as a representative macrophage cell line (32, 33), because the induction of monocytemacrophage differentiation by PMA accompanied by the flattening and adherence of cells to the surface of cell culture plates, the development of histological similarities to macrophages, and up-regulation of *MMP-2* and *MMP- 9* (13, 34). Previous studies have demonstrated that PMA activation of THP-1 cells stimulates expression of *TIMP- 1* and CD147 (34-36). Therefore, our *in vitro* model mimicked the activated inflammatory macrophages. As expected, our data indicated that *MMP-9* and *TIMP-1* mRNA and CD147 expressed at low levels in unstimulated THP-1 cells, whereas PMA-differentiated THP-1 cells increased their expression levels. Furthermore, expression of *MMP-2* and *TIMP-2* mRNA showed no significant changes.

CFS containing secreted bioactive compounds from *L. acidophilus* and LGG reduced *MMP-9* expression and activity, decreased the cell surface expression of CD147, and increased *TIMP-1* expression. To our knowledge, this is the first report indicating that secreted bioactives from *L. acidophilus* and LGG can modulate *MMP-9*, CD147 and *TIMP-1* in an inflammatory activated macrophage model *in vitro*. MMP-2 (Gelatinase-A) and *MMP-9* (Gelatinase-B) belong to gelatinase subgroup of MMPs and possess proteolytic activity to degrade extracellular matrix components such as gelatins, collagens, and laminin (37). We focused on MMP-2 and MMP-9 as increased MMPs during inflammatory processes. Treatment with CFS from LGG and *L. acidophilus* resulted in a significant decrease in *MMP-9* gene expression and activity, while it could not significantly decrease *MMP-2* gene expression and activity. Therefore, we speculated that decreased activity of MMP-9 may be due to reduced expression, protein synthesis, or secretion of MMP-9.

MMPs are activated after being cleaved extra-cellularly (38). *TIMP-1* and *TIMP-2* are two tissue inhibitors of MMP-9 and MMP-2, respectively (39). Our data indicated that CFS from LGG and *L. acidophilus* had the potential to increase *TIMP-1* expression, suggesting that decreased activity of MMP-9 was due to decreased protein synthesis or reduced conversion of pro-MMP-9 to active MMP-9 through increased expression of TIMP-1 as an inhibitor of MMP-9 activity, mediated by secreted bioactive compounds from LGG and *L. acidophilus*. In addition, since *TIMP-2* directly inhibited MMP-2 activity, CFSs did not affect *TIMP-2* expression. Therefore, MMP- 2 activity remained unchanged.

EMMPRIN/CD147 (extracellular matrix metalloproteinase inducer), a member of the immunoglobulin superfamily (40, 41), is known to induce expression and activity of several MMPs during inflammatory damages and wound healing (14-16). A significant decrease in cell surface expression of CD147 was observed in PMA-differentiated THP-1 cells treated with either *L. acidophilus* CFS or LGG CFS. According to our data in the present study, we speculated that the down-regulation of the expression and gelatinolytic activity of MMP-9 may be due to the inhibition of CD147, up-regulation of TIMP-1 expression and/or direct role of MMP-9. Another study has also indicated that *L. rhamnosus* and *Bifidobacterium breve (B. breve)* significantly suppress the ability of cigarette smoke-induced inflammatory mediators expression in human THP-1 macrophages through the suppression of *nuclear* factor-*kappa* B (NF-κB) activation (42). In addition, a number of studies have reported that L. *acidophillus* and LGG inhibit the activation of NF-κB by preventing the degradation of inhibitory kappa B alpha (IκBα) (43-48). Since MMP-9 is a target gene of NF-κB (49, 50) and an NF-κB response element exists in the CD147 promoter (51), suppression of NF-κB activation may be a possible pathway for inhibiting MMP-9 and CD147 expression mediated by* L. acidophilus* and LGG cell free supernatants.

## Conclusion

Our study indicated that secreted factors from probiotic bacteria *L. acidophilus* and LGG targeted MMP-9, *TIMP- 1*, and CD147 to inhibit inflammatory processes. Thus, *L. acidophilus* and LGG may be attractive candidates for in vivo examination of their anti-inflammatory effects. Further studies, including the characterization and mechanisms of action of bioactive factors, may support the use of probiotic-containing functional foods and supplements as a dietary strategy to prevent and treat inflammatory diseases.
